# Emerging medical therapies for congenital adrenal hyperplasia

**DOI:** 10.12688/f1000research.17778.1

**Published:** 2019-04-02

**Authors:** Phyllis W. Speiser

**Affiliations:** 1 Pediatrics, Zucker School of Medicine at Hofstra-Northwell Health, Lake Success, New York, 11042-2062, USA

**Keywords:** congenital adrenal hyperplasia, treatment

## Abstract

Congenital adrenal hyperplasia has traditionally been treated with daily oral doses of glucocorticoids and mineralocorticoid supplements. Such therapy does not precisely replicate the adrenal cortex's circadian pattern. As a consequence, patients are intermittently overtreated or undertreated leading to growth suppression in children, excess weight gain and altered metabolism. Several new treatments are on the horizon. This article will summarize some new potential therapies as adjuncts to, or replacement for, standard therapy.

## Introduction

Congenital adrenal hyperplasia (CAH) is caused by one of several inherited enzyme deficiencies. The most common form of the classic disorder, found in about 1:14,000 to 1:18,000 births, is steroid 21-hydroxylase deficiency. Mutations in *CYP21A2* (P450c21) impair adrenocortical production of cortisol and frequently aldosterone and lead to the accumulation of adrenal sex steroids ^1^. Allelic variation accounts for most phenotypic differences. Cardinal features of classic CAH include atypical development of the external genitalia in girls with manifest virilization. Both males and females have salt wasting with failure to thrive and potentially fatal hypovolemia and shock. Newborn screening, now universal in the US ^2^ and in many developed countries ^3^, can mitigate these complications (reviewed in 4). Despite life-saving glucocorticoid (GC) and mineralocorticoid (MC) oral therapies, treatment does not precisely replicate adrenal physiology. Individuals with CAH commonly experience adverse outcomes in terms of growth, metabolic, reproductive, and mental health endpoints ^5, 6^. This discussion of emerging medical treatments will be restricted to the classic or severe forms of steroid 21-hydroxylase deficiency.

## Improved glucocorticoid delivery

Normal adrenocortical secretion has a circadian rhythm quite distinct from that of blood cortisol levels achieved by administering two or three daily oral doses of GC medication ^7, 8^. Hydrocortisone (HC) subcutaneous delivery for 6 months via a programmed pump in eight adults with classic CAH produced significant reduction in adrenal androgens with improvement in quality of life and fatigue ^9^. Though conceptually attractive and perhaps applicable to highly motivated patients who are inadequately managed by conventional treatment, pump management is complex. An early trial with a once-daily modified-release oral HC preparation (Chronocort, Diurnal, Cardiff, UK) given to 16 adults with classic CAH decreased adrenal androgen precursors despite a slightly reduced daily HC dose ^10^. However, subsequent phase 3 trials apparently failed to demonstrate superiority to standard HC treatment and this potential new treatment is currently on hold. A different type of modified-release GC (Plenadren, Shire, London, UK) is approved in Europe for adrenal insufficiency but has not been formally tested in CAH.

In the US, the lowest-dose HC tablet is 5 mg, and in Europe 10 mg, excessive for infants and young children. Availability of pediatric-dose formulations would eliminate concerns about improper compounding of HC from tablets ^11, 12^. Based on favorable trial results ^13^, the European Medicines Agency has approved very-low-dose HC 1 mg granules (Alkindi, Diurnal) for treatment of adrenal insufficiency or CAH in infants and children. A US Food and Drug Administration new drug application is said to be pending.

## Androgen/estrogen antagonists and synthesis inhibitors

To ameliorate the effects of adrenal androgen excess, females with CAH often need treatment additional to GC replacement. Such treatments may include dermatologic therapies for acne and hirsutism or additional hormone treatments (or both) to regulate menses or aid conception. All steroidogenic pathways to androgens and estrogens depend on activity of the enzyme 17-hydroxylase/17,20-lyase (P450c17, CYP17A1). Abiraterone acetate is an orally active, potent P450c17 inhibitor ^14^ indicated for treatment of castration-resistant prostate cancer ^15, 16^. Short-term adjunctive treatment with 250 mg/day abiraterone acetate (alongside standard steroid replacement) normalized the pre-dose serum androstenedione levels in all six women with poorly controlled classic CAH ^17^. Because abiraterone acetate also inhibits gonadal steroid production and could be teratogenic, its use in CAH would be limited to pre-pubertal children, women using contraceptives, or men who receive gonadal replacement. A clinical trial is under way in pre-pubertal children with CAH (ClinicalTrials.gov Identifier: NCT02574910) with the goal of minimizing exogenous GC and endogenous adrenal sex steroid hormone exposure in order to normalize growth and pubertal development.

## Growth-promoting drugs

A systematic review and meta-analysis of adult height in individuals with classic CAH diagnosed before the age of 5 years included just over 1000 children in 35 studies that met the eligibility criteria ^18^. The pooled data indicated a corrected adult height standard deviation (SD) of −1.0. The average heights were 169 cm (66.5 inches) for men and 157 cm (61.8 inches) for women, both within the normal range for shorter than average adults in the general population. These data obviate the routine use of growth-promoting medications that are considered only for individuals whose heights were expected to be at least −2.25 SDs. Subgroup analysis revealed that the addition of early MC treatment was associated with increased height outcome ^18^.

A 2001 report tested growth hormone alone (n = 12) or in combination with leuprolide acetate (n = 8) to enhance growth in CAH patients with evidence of early puberty. Follow-up over 2 years showed improved predicted adult height, but as of this date, no data have been published to document actual adult heights ^19^. A proof-of-concept trial demonstrated that co-administration of growth hormone plus an aromatase inhibitor (again, alongside standard steroid replacement) improved adult height in a single adolescent male patient with CAH ^20^.

Since normal adult height may be achieved through judicious use of standard GC and MC therapies, further long-term prospective randomized and carefully controlled studies are needed to determine whether the use of growth-promoting drugs is safe and cost-effective in individuals with CAH. At present, such treatments are not considered standard care in children with CAH.

## Other medical strategies

Reducing adrenocorticotropic hormone (ACTH) production is another mechanism for minimizing adrenal androgen excess. In a small trial of eight women with classic CAH, the selective corticotropin-releasing hormone receptor type 1 antagonist, NBI-77860, was added to conventional therapy ^21^, resulting in a more than 40% reduction in the morning ACTH surge and about 27% lower serum 17OHP. Variable reductions of androstenedione and testosterone were observed.

Mitotane, a different type of adrenolytic used for treatment of adrenocortical cancer and Cushing syndrome, was administered to a man with classic CAH and testicular adrenal rest tumors (TARTs) who was infertile for 2 years ^22^. Adrenal androgen precursors were suppressed and TARTs regressed. Paternity was achieved following an increase in sperm count. Mitotane is a potential teratogen (pregnancy category D) and induces CYP3A4, increasing GC clearance, and therefore is not considered a useful adjunct to CAH therapy. ATR-101 (ClinicalTrials.gov Identifier: NCT02804178), which inhibits acyl co-A cholesterol acyltransferase and shares some mechanisms with mitotane ^23– 25^, has been tested in adults with classic CAH; results of this trial have not yet been published.

## Adrenalectomy

Adrenalectomy reduces virilization in females and permits decreased GC doses but this is considered a rather radical approach because of surgical risk. Moreover, there is an increased risk of life-threatening adrenal crisis with absolute dependence on exogenous hormone replacement and loss of potentially beneficial hormones—for example, dehydroepiandrosterone (DHEA) and epinephrine—from the adrenal medulla. A final consideration is that adrenalectomy may inadvertently cause the development of gonadal adrenal rest tumors that secrete androgens ^26, 27^. For these reasons, the initial enthusiasm has been tempered by long-term complications. Individuals who are known to be non-adherent are poor candidates for adrenalectomy. A systematic review of bilateral adrenalectomy in CAH ^28^ identified 48 cases ranging from infancy to adulthood and most were carried out for uncontrolled androgen excess or iatrogenic Cushing syndrome (or both) caused by administration of large GC doses to achieve control. Post-operative amelioration of these symptoms was noted in most patients, including three women who were able to conceive following adrenalectomy. In contrast, about 40% of patients experienced adverse outcomes, including eight patients with adrenal crisis and one death in an infant. Five males developed adrenal rest tumors requiring surgical removal. Unexpectedly, two males had regression of TARTs ^28^. The conclusion from this review is that adrenalectomy is effective for relief of refractory adrenal androgen excess, but that candidates for adrenalectomy must be chosen judiciously and educated extensively regarding post-operative risks.

## Epinephrine deficiency

Individuals with classic CAH have adrenomedullary insufficiency because GCs are required for development and regulation of the adrenal medulla ^29^. The physiologic responses of glucose, insulin, and leptin pathways are dysregulated during exercise among adolescent patients lacking both cortisol and epinephrine ^30, 31^. The clinical implications of epinephrine deficiency are not fully known, but it may contribute to hypoglycemia during febrile illnesses, especially in young children, and impair the response to stress ^32, 33^. Decreased epinephrine production has been observed in newborns with classic CAH compared with controls; norepinephrine levels were similar ^32^. Epinephrine replacement or supplementation has not been studied. It is not known whether a compensatory norepinephrine response is sufficient.

## Gene therapy

In two decades since the initial report that adenoviral gene therapy transiently restored enzyme activity in a mouse model of 21-hydroxylase deficiency ^34^, there have been no human trials. Animal research is ongoing, and intravenous injection of an adenoviral-Cyp21a1 vector in such mice allowed functional enzyme expression in adrenal tissue, resulting in weight gain, near normal progesterone levels, and improved stress response for more than 15 weeks ^35^. However, in another laboratory setting, the therapeutic effect lasted only 8 weeks ^36^. Auto-transplantation of Cyp21a1-expressing fibroblasts into 21-hydroxylase–deficient mouse subcutaneous tissue or direct injection of adenovirus-Cyp21a1 constructs into mouse muscle demonstrated enzyme efficacy for about 4 weeks ^37^. Thus, both adrenal and extra-adrenal induction of Cyp21a1 can temporarily ameliorate steroid metabolism in 21-hydroxylase null mice. It is unclear whether the murine data will be translated into effective human treatments. Permanent correction of mutations causing CAH with gene therapy directed at a patient’s own adrenal stem cells would theoretically cure CAH and supplant imperfect steroid replacement. Cell-based therapies and gene-editing technology now in development may be options for disease cure in the future ^38^.
