# Supplementary material for: Emerging medical therapies for congenital adrenal hyperplasia
Source: F1000Res. 2019 Apr 2;8:363. [Version 1] doi: 10.12688/f1000research.17778.1 (PMC6446492; doi:10.12688/f1000research.17778.1)
Supplement: Peer review history

# Peer review: Reviewer response for version 1

**DOI:** 10.5256/f1000research.19438.r46296

**Published:** 2019-04-02

**Authors:** Peter C Hindmarsh

**Affiliations:** 1 Institute of Child Health, University College London, London, UK

I have read this submission. I believe that I have an appropriate level of expertise to confirm that it is of an acceptable scientific standard.

# Peer review: Reviewer response for version 1

**DOI:** 10.5256/f1000research.19438.r46295

**Published:** 2019-04-02

**Authors:** Maria G Vogiatzi

**Affiliations:** 1 Division of Endocrinology and Diabetes, Children's Hospital of Philadelphia, Philadelphia, PA, USA

I have read this submission. I believe that I have an appropriate level of expertise to confirm that it is of an acceptable scientific standard.

# Peer review: Reviewer response for version 1

**DOI:** 10.5256/f1000research.19438.r46297

**Published:** 2019-04-02

**Authors:** Roberto Salvatori

**Affiliations:** 1 Division of Endocrinology, Diabetes, and Metabolism, Johns Hopkins University, Baltimore, Maryland, USA

I have read this submission. I believe that I have an appropriate level of expertise to confirm that it is of an acceptable scientific standard.

# Peer review: Reviewer response for version 1

**DOI:** 10.5256/f1000research.19438.r46298

**Published:** 2019-04-02

**Authors:** Anna Nordenström

**Affiliations:** 1 Department of Women's and Children's Health, Karolinska Institutet, Stockholm, Sweden

I have read this submission. I believe that I have an appropriate level of expertise to confirm that it is of an acceptable scientific standard.
